# Torsion of a Subserosal Myoma Managed by Gasless Laparoendoscopic Single-Site Myomectomy with In-Bag Manual Extraction

**DOI:** 10.1155/2016/7831270

**Published:** 2016-03-13

**Authors:** Akihiro Takeda, Hiromi Nakamura

**Affiliations:** Department of Obstetrics and Gynecology, Gifu Prefectural Tajimi Hospital, 5-161 Maebata-cho, Tajimi, Gifu 507-8522, Japan

## Abstract

Acute torsion of a subserosal myoma is a rare surgical emergency that is infrequently diagnosed preoperatively. Furthermore, laparoendoscopic single-site (LESS) myomectomy with in-bag tissue extraction for the management of this disorder has not yet been described. A 43-year-old, gravida 1, para 1 woman was referred to our department due to a solid pelvic mass associated with persistent abdominal pain. A pedunculated subserosal myoma with torsion was strongly suspected based on ultrasonography and magnetic resonance imaging. Emergency LESS surgery showed that the subserosal myoma arising from the posterior uterine wall torted at its pedicle in the cul-de-sac. The twisted myoma node was excised by coagulation and cut using a LigaSure Atlas, followed by in-bag manual morcellation and extraction through an umbilical wound. The present case report emphasizes that LESS myomectomy with in-bag tissue extraction is a feasible minimally invasive surgical option for the management of subserosal myoma with torsion after a precise imaging-based diagnostic evaluation.

## 1. Introduction

Uterine myoma is the most common benign neoplasm in women of reproductive age [1]. The symptoms of a uterine myoma include menorrhagia, abnormal bleeding, dysmenorrhea, dyspareunia, pelvic pain, and frequent urination [1]. Although most of these symptoms occur as chronic manifestations, myoma can cause acute complications in rare cases [1, 2]. Torsion of a pedunculated subserosal myoma is one of the causes of significant morbidity with intense abdominal pain and is recognized as a surgical emergency [3–10]. Once a delay in the diagnosis and surgical intervention occurs, ischemic gangrene of the torted myoma may develop, followed by a severe infection, such as peritonitis [3].

While surgical exploration of the twisted myoma usually resolves the problem, the challenge is in making the appropriate diagnosis, which is difficult because of the limited known imaging-based diagnostic characteristics [3, 5, 8]. As a result, its diagnosis is most frequently made at the time of surgical exploration [2].

For the management of acute torsion of myoma, laparoscopic myomectomy has recently evolved as a feasible minimally invasive surgical option [5, 6, 8–10] and is replacing the traditional laparotomic approach [4, 7]. However, the use of laparoendoscopic single-site (LESS) myomectomy [11, 12], which has a potential cosmetic advantage over multiport laparoscopic surgery, has not yet been described as a treatment for this disorder.

We herein report a rare case of torsion of a subserosal myoma that was successfully managed by emergency LESS myomectomy with transumbilical in-bag manual morcellation and extraction, after a diagnosis was made based on ultrasonography and magnetic resonance imaging (MRI). Institutional review board approval from Gifu Prefectural Tajimi Hospital was obtained to report this case.

## 2. Case Report

A 43-year-old, gravida 1, para 1 woman without any previous disease history presented to her gynecologist with complaints of lower abdominal pain and was managed conservatively as a pelvic infection without diagnosis of subserosal myoma. Six months later, she was transferred to an emergency department of a hospital due to recurrent severe lower abdominal pain. The white blood cell count and C-reactive protein value at admission were elevated to 9,900/*µ*L (normal value: 4,000–10,000/*µ*L) and 4.9 mg/dL (normal value < 0.3 mg/dL), respectively. Since the* Chlamydia trachomatis* antibody testing was positive, minocycline was administered intravenously under a diagnosis of pelvic inflammatory disease.

**Figure 1:**
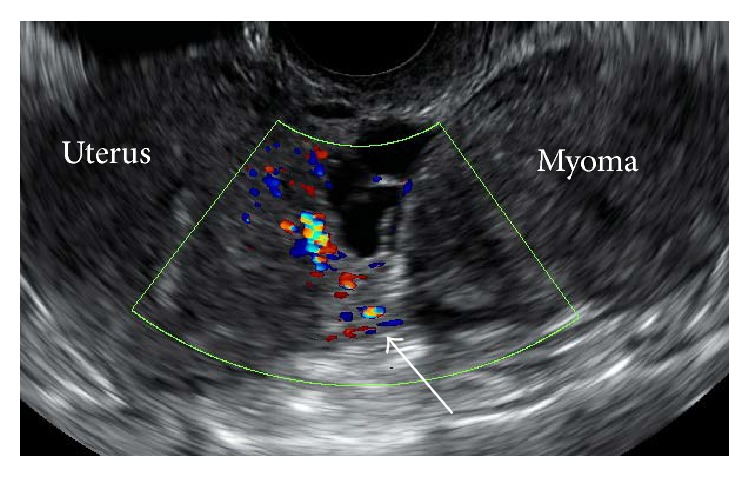
Transvaginal color Doppler ultrasonography showing a pedunculated myoma connected to the uterine body. The blood flow was interrupted at the base of the myoma pedicle (arrow), suggesting the occurrence of torsion.

**Figure 2:**
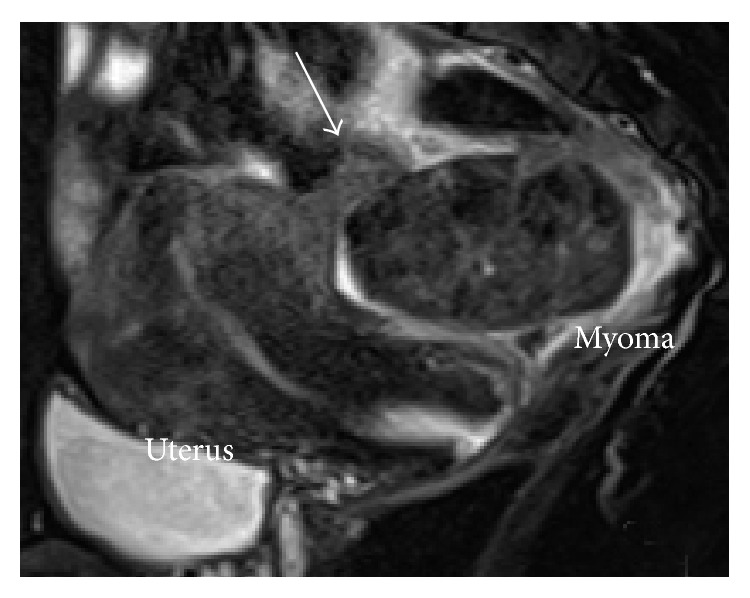
Sagittal T2-weighted MRI showing a torted pedicle of the subserosal myoma (arrow).

Five days later, she was referred to our department because of persistent abdominal pain. By a vaginal examination, a tender solid mass was palpated in the cul-de-sac. On transvaginal ultrasonography, a solid pedunculated mass measuring 55 × 41 mm was identified (Figure 1). Since the bilateral ovaries showed a normal appearance, this solid mass was strongly suspected to be a subserosal myoma. Further evaluation by color Doppler ultrasonography showed that the blood flow was interrupted at the base of the pedicle of this mass (Figure 1, arrow), suggesting the presence of torsion. Sagittal T2-weighted MRI (Figure 2) confirmed this finding by showing a subserosal myoma with a torted pedicle (Figure 2, arrow).

**Figure 3:**
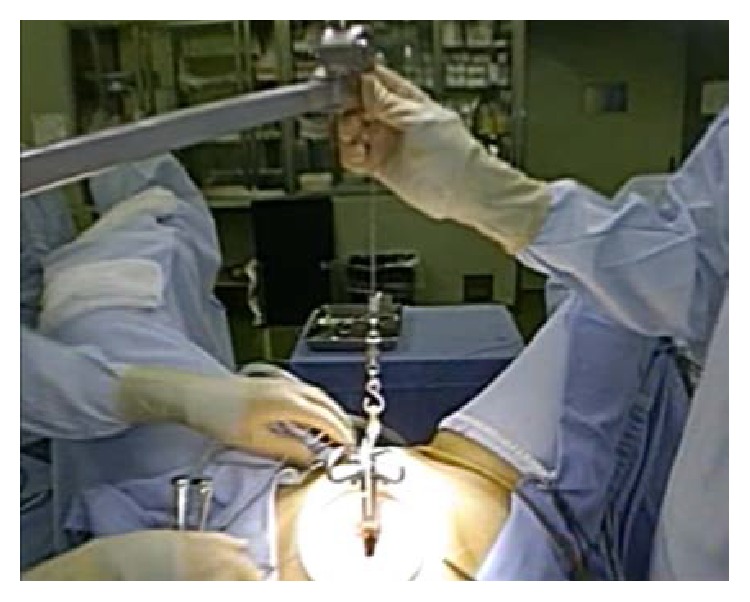
Gasless laparoendoscopic single-site myomectomy was performed through an Alexis wound retractor (small size, Applied Medical, Rancho Santa Margarita, CA) attached via a 2.5 cm midline umbilical skin incision with the surgical view secured by abdominal-wall lifting with an intra-abdominal fan retractor system (Mizuho Co., Tokyo, Japan).

Under a diagnosis of a pedunculated subserosal myoma with torsion, gasless LESS surgery was performed through an Alexis wound retractor (small size, Applied Medical, Rancho Santa Margarita, CA) attached via a 2.5 cm midline umbilical skin incision with the surgical view secured by abdominal-wall lifting with an intra-abdominal fan retractor system (Mizuho Co., Tokyo, Japan) (Figure 3) [13].

**Figure 4:**
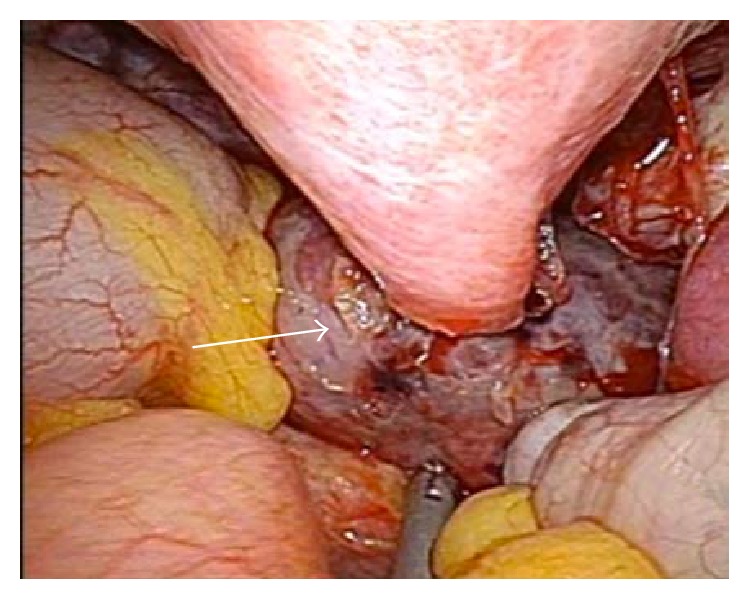
A single-port laparoscopic view showing the necrotized subserosal myoma with torsion at its pedicle in the cul-de-sac (arrow).

Under laparoscopic vision using a rigid 5 mm, 30-degree EndoEYE laparoscope (Olympus, Tokyo, Japan), a necrotized subserosal myoma with torsion at its pedicle was identified in the pouch of Douglas (Figure 4, arrow). Furthermore, adhesion of the myoma surface to the left adnexa and sigmoid colon was identified. After adhesiolysis by blunt dissection, the subserosal myoma was mobilized, followed by coagulation and cutting of its pedicle using a LigaSure Atlas (Covidien Japan, Tokyo, Japan) with minimum hemorrhage.

**Figure 5:**
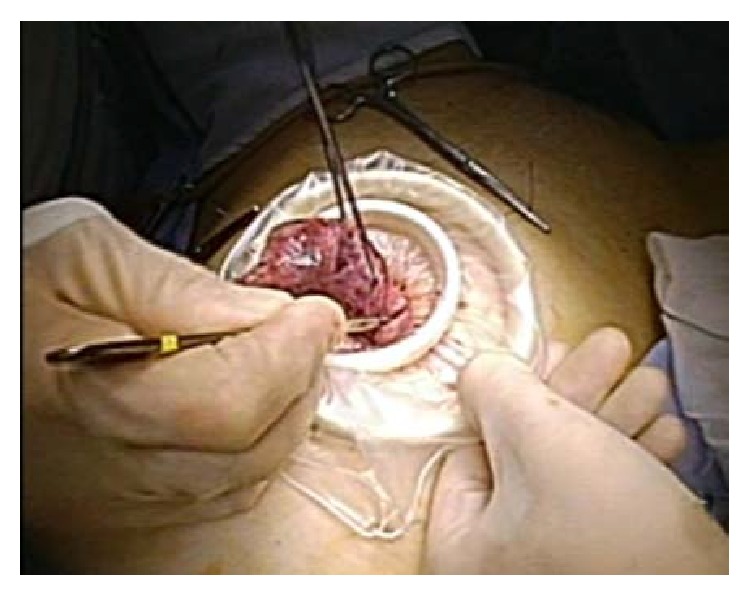
Extracorporeal extraction of excised myoma was performed through the umbilical incision by in-bag manual morcellation using a number 11 surgical blade.

**Figure 6:**
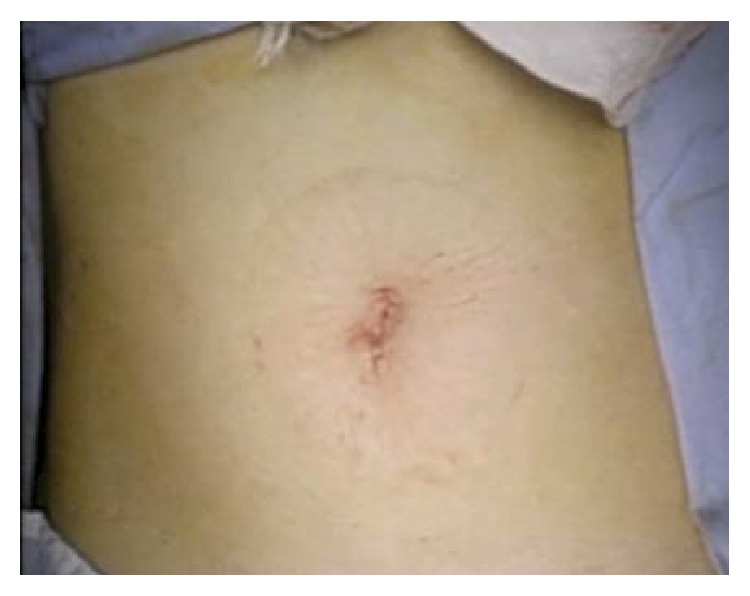
The cosmetic outcome of the wound, which was concealed in the umbilicus after gasless laparoendoscopic single-site myomectomy.

After the excised myoma was put into an Endocatch II retriever bag (Covidien Japan), the thread of the retriever bag was pulled outside of the abdomen via the umbilical minilaparotomic incision and the edges of the pouch were exteriorized. For efficient retrieval, another smaller Alexis wound retractor (X-small size, Applied Medical) was placed inside the retriever bag to maximize the size of the opening as well as fixing unevenness in the circumference (Figure 5) [14]. Then, the myoma tissue was manually morcellated by a number 11 surgical scalpel blade in the bag to prevent tissue dissemination in the peritoneal cavity. The peritoneal cavity was rigorously washed with saline and the uterine wound was covered by Interceed (Ethicon, Somerville, NJ) to prevent adhesion formation. After the surgical procedure was completed, the wound retractor was removed and the umbilical wound was closed (Figure 6). The length of the surgery was 37 minutes and the excised tissue weight was 62 g. The postoperative course was uneventful.

## 3. Discussion

Among uterine myomas, which are by far the most common gynecological tumors, subserosal myomas located beneath the uterine serosa are the second most common, after the intramural type [3]. Although subserosal myomas are usually asymptomatic [1, 2], they may manifest as an abdominal emergency in limited cases, particularly when they undergo torsion around their axis after becoming pedunculated [1, 2].

The severity of symptoms varies, depending on the degree of the rotation and the speed at which the torsion develops. If the torsion is partial and intermittent with spontaneous untwisting, the symptoms may subside, become constant, or spontaneously resolve, as might have occurred after the first event in the present case [3]. In contrast, if the torsion of the pedicle is complete, as what occurred during the second event in this case, it results in circulatory stasis that is initially associated with venous edema and congestion, followed by compression of the arterial blood supply that resolves as the torsion and the resultant edema progress [3].

If torsion of the myoma would be left untreated, hemorrhagic infarction can cause severe necrosis of the involved myoma, followed by infection, and then peritonitis. This is the most severe and ominous form of leiomyoma degeneration [3]. Therefore, although the condition is relatively uncommon, acute torsion of a subserosal myoma should be recognized as a surgical emergency requiring a precise and prompt diagnosis.

The diagnosis of an acute torsion of subserosal myoma could be made based on the clinical manifestations and diagnostic imaging examinations [8]. The diagnostic value of color Doppler ultrasonography [8], MRI [5, 8], and computed tomography [3] has previously been advocated. However, the definitive diagnosis based on the imaging findings may be usually difficult preoperatively, partly because specific findings associated with this condition have not yet been clarified due to its rarity [3, 5, 8].

In this clinical context with respect to a pelvic mass, adnexal torsion should be considered first. However, demonstration of normal bilateral adnexa by ultrasonography can easily exclude the presence of adnexal torsion [3] as in the present case. Furthermore, if real-time transvaginal color Doppler ultrasonography can demonstrate a twisted pedicle with interruption of the blood flow at its base, in addition to a larger distance between the myoma and the uterus, it strongly indicates the occurrence of torsion of a subserosal myoma. As has been reported in previous studies and shown in the present case report, MRI can also have supplementary diagnostic value when a twisted pedicle can be recognized between the uterine corpus and subserosal myoma [5].

Once the diagnostic imaging evaluation indicates the presence of torsion of a subserosal myoma, emergency surgical exploration should be considered to avoid the significant morbidity caused by progression of tissue necrosis. Although laparoscopic myomectomy [5, 6, 8–10] can be a feasible minimally invasive surgical option compared with laparotomy [4, 7], multiple abdominal incisions ranging from 5 to 12 mm in length are usually required in traditional gynecological laparoscopic surgical procedures to accommodate the working ports for the insertion of the laparoscope and surgical instruments.

As a potentially less traumatic surgical method with cosmetic advantages, transumbilical LESS surgery was recently introduced for various types of gynecological surgery, including myomectomy [11, 12]. Although feasibility of LESS myomectomy has been shown in previous studies [11, 12], its incorporation into standard surgical technique is still limited because of its technical difficulties, such as the limited motion and potential for clashes between instruments [11]. In the present case, the needs for intracorporeal suturing and tying, which can make LESS myomectomy more challenging, could be avoided, since hemostasis was obtained by coagulation with a bipolar vessel sealing system. Thus, in selected cases of subserosal myoma, LESS myomectomy could be a feasible surgical option with cosmetic advantages over conventional multiport laparoscopic myomectomy.

Previous report indicated that gasless single-port laparoscopy with a wound retractor has potential benefits over pneumoperitoneum laparoscopy [15]; since gas leakage is not a major concern in gasless LESS procedure, a variety of intracorporeal and/or extracorporeal surgical procedures can be performed more easily even by gynecologic surgeons with standard laparoscopic skills. Furthermore, in the present case, a single large umbilical incision permits free access to the abdominal cavity, allowing efficient removal of a solid myoma tissue, compared with multiport laparoscopy utilizing 5 to 12 mm ports.

For the retrieval of an excised myoma during laparoscopic myomectomy, fragmentation of the myoma tissue is required to remove it out of the body through the minimal incision [16]. To achieve this, morcellation by either a mechanical power morcellator or a surgical scalpel in the peritoneal cavity should be included. However, if this morcellation procedure is performed in the open peritoneal space, it raises significant concerns about the dissemination of myoma particles, which can subsequently develop into parasitic leiomyomatosis in the peritoneal cavity after implantation and growth [16].

To avoid this potentially health-threatening issue, a number of procedures that can be used to morcellate the myoma tissues under closed conditions have recently been advocated [14, 17]. In the present case, in-bag closed manual morcellation through an umbilical incision was utilized as an easy and safe method [14]. After dilating the umbilical incision with a wound retractor, manual morcellation using a number 11 surgical scalpel blade [14] allowed us to efficiently extract the myoma tissue without any fear of the dissemination of even minute myoma particles.

## 4. Conclusion

The present case report emphasizes that LESS myomectomy with in-bag tissue extraction is a feasible minimally invasive surgical option for the management of a pedunculated subserosal myoma with torsion after a precise diagnostic evaluation imaging is performed.
